# Supplementary material for: Semi-automated isolation of parenchymal and non-parenchymal liver cells from mice and humans with enhanced stellate cell fraction
Source: Cell Biosci. 2026 Mar 14;16:45. doi: 10.1186/s13578-026-01554-7 (PMC13123112; doi:10.1186/s13578-026-01554-7)
Supplement: Supplementary file 1 — Supplementary Material 1. [file 13578_2026_1554_MOESM1_ESM.docx]

**Supporting Materials**

**Semi-automated isolation of parenchymal and non-parenchymal liver cells from mice and humans with enhanced stellate cell fraction**

Anne Dropmann^1*^, Bedair Dewidar^1,2*^, Kerry Gould^1*^, Raquel L. Baccetto^1^**,** Christoph Meyer^1^, Tiziana Caccamo^1^, Pia Erdoesi^1^, Şamil Ayvaz^1^, Andrea Scheffschick^3^, Georg Damm^3^, Daniel Seehofer^3^, Laura Kim Feiner^4^, Bianca Kruse^4^, Claudia Rubie^4^, Matthias Glanemann^4^*,* Vanessa Orth^5^, Emrullah Birgin^6^, Nuh Rahbari^6^, Matthias P. Ebert^7,8,9^, Steven Dooley^1^, Seddik Hammad^1, #^

^1^Molecular Hepatology Section, Department of Medicine II, Medical Faculty Mannheim, Heidelberg University, 68167 Mannheim, Germany

^2^Department of Pharmacology and Toxicology, Faculty of Pharmacy, Tanta University, 31527 Tanta, Egypt

^3^Department of Hepatobiliary Surgery and Visceral Transplantation, University Hospital, Leipzig University, Leipzig, Germany

^4^Department of General, Visceral, Vascular and Pediatric Surgery, Saarland University, Homburg/Saar, Germany

^5^Department of Surgery and European Center of Angioscience (ECAS), Medical Faculty Mannheim, Heidelberg University, Theodor-Kutzer-Ufer 1-3, 68167, Mannheim, Germany

^6^Department of General and Visceral Surgery, University Hospital Ulm, Albert-Einstein-Allee 23, 89081, Ulm, Germany

^7^Department of Medicine II, Medical Faculty Mannheim, Heidelberg University, Theodor-Kutzer-Ufer 1-3, 68167, Mannheim, Germany

^8^DKFZ-Hector Cancer Institute at the University Medical Center, Im Neuenheimer Feld 280, 69120, Heidelberg, Germany

^9^Molecular Medicine Partnership Unit, European Molecular Biology Laboratory, Meyerhofstraße 1, 69117, Heidelberg, Germany

*shared first authorship

#Corresponding author:

Email: [seddik.hammad@medma.uni-heidelberg.de](mailto:seddik.hammad@medma.uni-heidelberg.de)

**Materials and methods**

**Cell yield and viability**

Cell density and viability were assessed using the MACSQuant Analyzer 10 (Miltenyi Biotec, Germany, cat. no. 130-096-343) with propidium iodide (PI, Miltenyi Biotec, Germany, cat. no. 130-093-233) incorporation. To determine cell number and viability, Trypan Blue (Sigma Aldrich, Germany, cat. no. T8154) exclusion assays were performed. Briefly, equal volumes of cell suspension and Trypan Blue solution (0.4% v/v) were mixed and examined under a bright-field microscope (Leica Microsystem, Germany) using a Neubauer cell counting chamber (neoLab Migge, Germany, cat. no. C-1003). The percentage of viable and non-viable cells was then calculated.

**Immunofluorescence staining and purity of separated liver cells**

To assess the purity of isolated cells, immunofluorescence staining was performed using F4/80, LYVE1, and Albumin antibodies as markers for KC, LSEC, and HC, respectively (Supporting Table 1). Desmin and Acta2 (α-SMA) antibodies were used to quantify total HSC and activated HSC populations. Additionally, CD271 protein expression was evaluated for confirmation (Supporting Table 1). Primary cells were seeded at 1×10³ cells/well in 8-well chamber slides (Corning, Germany, cat. no. 354118) with DMEM medium supplemented with 10% FBS, 1% P/S, and 1% L-glutamine. After 24 hours, the medium was removed, and cells were fixed with 4% paraformaldehyde (ROTI Histofix 4%, Carl Roth, Germany, cat. no. P087.1) for 10 minutes at room temperature (RT). Fixed cells were washed twice with PBS and incubated with 3% bovine serum albumin (BSA, Serva, Germany, cat. no. 11930.03) in PBS for 45 minutes at RT. Cells were then incubated with primary antibodies (Supporting Table 1) diluted in 0.3% BSA/PBS for 2 hours at RT. After washing, cells were incubated with fluorescence-labeled secondary antibodies (Supporting Table 3) for 1 hour at RT. Nuclei were counterstained with DRAQ5 (1:1000, New England Biolabs, Canada, cat. no. 4084) and examined using a Leica TCS SP8 confocal microscope (Leica Microsystems, Germany). To quantify cell purity, image analysis was performed using ImageJ (<https://imagej.nih.gov/ij/>). First, image channels were split into nuclear staining (DRAQ5) and specific cell marker channels (e.g., Desmin for HSC). Total nuclei were counted in the DRAQ5 channel using the “Analyze Particles” tool with adjusted settings for cell size. Next, the threshold for specific cell marker channels was optimized to enhance the signal, and a binary mask was created. Images were merged, converted to an 8-bit black-and-white format, and analyzed for overlaying signals by adjusting the threshold. Positive cells were quantified using the “Analyze Particles” tool. Purity was calculated as the percentage of cell expressing specific cell markers relative to total DRAQ5 positive nuclei. On average, at least 2000 cells per condition were analyzed.

**MACS Flow Cytometry**

Freshly isolated CD271^+^ cells (based on the magnetic bead-labeled protocol described above) were suspended in 2 ml of MACS buffer (Miltenyi Biotec, Germany, cat. no. 130-092-747) and centrifuged for 10 minutes at 300 g. The cell pellets were resuspended in 100 µl of MACS buffer and fixed with 1 ml of 4% paraformaldehyde (PFA) for 15 minutes at RT. Next, cells were washed with PBS and permeabilized for 15 minutes with 0.5% Tween 20 (neoFroxx, Germany, cat. no. 1247LT001). To block non-specific binding of antibodies, cells were incubated with 10 µl of Fc-receptor blocker (Miltenyi Biotec, Germany, cat. no. 130-092-575) for 5 minutes at 4°C. After washing with PBS, cells were incubated in the dark with primary antibodies, including CD271 and Desmin (Supporting Table 1), dissolved in MACS buffer with 0.5% Tween 20. After 1 hour, cells were washed twice with PBS and incubated with secondary antibodies for 30 minutes in the dark (Supporting Table 3). Finally, cells were washed twice with PBS, and cell pellets were resuspended in 100 µl of MACS buffer. Immediately before MACS flow cytometry, cells were incubated with propidium iodide (PI, 1:100). Analysis was performed using the MACSQuant Analyzer 10, and cell debris and PI-positive cells were gated out using MACSQuantify Software 2.13. The remaining cells were gated for CD271 and Desmin positivity both before and after AutoMACS separation to evaluate the enrichment and specificity of CD271 for hepatic stellate cell (HSC) isolation.

**IHC staining of human liver tissue**

Serial liver tissue sections (3–5 µm) were deparaffinized in xylene and rehydrated through graded ethanol dilutions. Antigen retrieval was performed in EDTA buffer (1 mmol/L, pH 8.0) for 10 minutes. Slides were then incubated with an endogenous peroxidase blocker (Dako, Germany, cat. no. S2023, ready-to-use) for 20 minutes at RT. After washing with PBS, sections were incubated overnight at 4°C with primary antibodies against CD271, or ACTA2 (Supporting Table 1). The following day, slides were washed with PBS and incubated with secondary antibodies for 1 hour at RT (Supporting Table 3). Brown staining was developed using diaminobenzidine (DAB; Merck, Germany, cat. no. 102924), and nuclei were counterstained with hematoxylin (Merck, Germany, cat. no. 115938). The slides were scanned shortly after the staining procedure using the slide scanner Aperio 8 (Leica).

**Co-immunofluorescence staining of liver tissue**

To identify and localize CD271-positive cell types in mouse and human liver tissue, co-immunofluorescence staining with CD271, Desmin, and ACTA2 antibodies was performed, following the standard protocol of manufacturers with minor modifications. After deparaffinization and rehydration, antigen retrieval was performed by incubating slides in 0.1 mM EDTA solution (pH 8.4, Sigma Aldrich, Germany, cat. no. E9884) and heating for 10 minutes in a microwave at 15-second intervals. After washing, slides were incubated with 3% BSA/PBS solution for 1 hour at RT to block non-specific binding.

Primary and secondary antibody incubations were performed as described above (Supporting Tables 1 and 3). Slides were counterstained with DRAQ5 and mounted for fluorescence imaging. The slides were scanned shortly after the staining procedure using the slide scanner Aperio 8 (Leica).

***In situ* hybridization (ISH) for CD271 and Desmin using RNAscope^©^ Multiplex combined with α-SMA (ACTA2) antibody in human and mouse tissues**

For the RNA in situ hybridisation the RNAscope Multiplex Fluorescent v2 Assay (ACDBio by Bio-Techne) was performed according to the manufacturer’s protocol, using the RNAscope Probe Hs-CD271-CDS and m-CD271-CDS (ACDBio by Bio-Techne) and TSA Vivid™ Fluorophore Kit 570/520/640 (TOCRIS by Bio-Techne) for signal development. Two additional slides were used as positive and negative control using RNAscope 3-plex Positive Control Probe-Hs and RNAscope 3-plex Negative Control Probe-Hs (ACDBio by Bio-Techne). A positive control probe, targeting human or mouse housekeeping genes, and a negative control probe, specific for bacterial RNA, was used to validate the signal in the stained patient or mouse samples. Only signal that resembled the signal on the positive control slide was evaluated as positive signal of CD271-RNA. Each bright dot indicates the presence of one or more molecules of RNA, which have been detected by the labeled probe after amplification. In top of this, we stained the livers of mouse or human with Desmin or ACTA2 antibodies as previously described (Supporting Tables 1 and 3). Additionally, we stained the liver of human with Desmin RNA probe (Supporting Table 4). Slides were counterstained with Fluoroshied with DAPI (F6057-20ml, Sigma), and mounted for fluorescence imaging. The slides were scanned immediately after the staining procedure using the Aperio 8 slide scanner (Leica).

**Cell culture and treatment**

Freshly isolated primary HSC were seeded at a density of 1.5-3 x 10^5^ cells/well in 6- or 12-well plates (Greiner Bio One, Germany, cat. no. 657160) with DMEM supplemented with 10% FBS, 1% P/S, and 1% glutamine. After 24 hours, the medium was replaced by a starvation medium, i.e. growth medium with 0.5% FBS, and cells were treated with 5 ng/ml transforming growth factor (TGF)-β1 (Peprotech, USA, cat. no. 100-21C) for 72 h, or with starvation medium only. Finally, protein and RNA lysates were collected for immunoblotting and RT-PCR at the day of isolation and 3 days after cultivation.

**Quantitative real-time (RT)-PCR**

After cytokine treatment, cultured cells were washed twice with PBS. Next, total RNA was isolated using the InviTrap Spin Universal RNA Mini Kit (Invitek Molecular, Germany, cat. no. 1060100200) or the Qiagen RNeasy Mini Kit (Cat No./ID: 74104) according to manufacturer’s instructions. RNA concentration was measured with the Infinite M200 microplate reader (Tecan, Switzerland). From 500 ng total RNA, cDNA was transcribed with RevertAid H Minus Reverse Transcriptase (Thermo Fischer Scientific, USA, cat. no. EP0451) and used for RT-PCR with PowerUP SYBR Green Master Mix (Life Technologies, USA, cat. no. A25918) in a StepOnePlus RT-PCR System (Applied Biosystems USA from Thermo Fisher Scientific, Germany, cat. no. 4376600). A list of the primer pairs used (Eurofins, Germany) is provided in Supporting Table 5. Ppia was the internal reference gene used. Target gene relative expression was determined with the ΔΔCt method, and a melt curve was created to ensure primer specificity. Each sample was measured in triplicate.

**Affymetrix gene array analysis**

For identification of genes expressed differentially in Nycodenz-isolated HSC, we used the Gene Expression Omnibus (GEO) of the National Center for Biotechnology Information (NCBI) previously reported gene array study (GSE34640) of Balb/C cells upon culturing with and without KC (1). To eliminate analytical bias that might arise from data reprocessing, the NCBI GEO2R tool was used to directly determine the differentially expressed genes between quiescent (freshly isolated) and FBS activated HSC for 64 hours. Gene annotation numbers were 1426732_at, 1416454_s_at and 1454903_at for Des (Desmin), Acta2 (α-Sma) and Cd271, respectively. We analyzed publicly available human samples across liver disease-related datasets (GSE84044), mouse samples E-MTAB-2445 (a single CCl_4_-injected liver; (2), GSE222576 (fibrotic liver by CCl_4_; (3), GSE37559 (Mdr2*KO* livers; (4) via Array Express (<https://www.ebi.ac.uk/biostudies/arrayexpress>) or NCBI's Gene Expression Omnibus (GEO) (<https://www.ncbi.nlm.nih.gov/geo/>). Data are presented as an expression value ± standard deviation.

**Immunoblotting**

Cells were lysed with radioimmunoprecipitation assay (RIPA) buffer (20 mM Tris-HCl, pH 7.5, 150 mM CaCl2, 1% sodium deoxycholate, 1% Nonidet P-40, 0.1% SDS, 1 mM EDTA, 0.5 mM EGTA), in presence of a Protease Inhibitor Cocktail (Sigma Aldrich, Germany, cat. no. P8340) and a Phosphatase Inhibitor Cocktail II (Sigma Aldrich, Germany, cat. no. P5726). The protein concentration of the cell lysates was determined with an Infinite M200 microplate reader (Tecan, Switzerland) using the DC Protein Assay Kit (Bio-Rad, Germany, cat. no. 5000112). Proteins (20-30 µg) were separated using 10% sodium dodecyl sulfate-polyacrylamide gels (Thermo Fisher Scientific, Germany, cat. no. NP0336BOX). After electrophoresis, proteins were transferred from gels to Amersham Protran nitrocellulose membranes (Carl Roth, Germany, cat. no. 4685.1) and blocked with 5% non-fat milk (Carl Roth, Germany, cat. no. T145.1) in Tris-buffered saline with Tween 20 (TBST buffer: 10 mM Tris, pH 8.0, 150 mM NaCl, and 0.5% Tween 20) for 2 hours at RT. Afterwards, the membranes were incubated overnight at 4°C with primary antibodies (Supporting Table 1). The next day, membranes were washed with TBST buffer and incubated with secondary antibodies for 1 hour at RT (Supporting Table 3). Finally, membranes were developed with Pierce™ enhanced chemiluminescent substrate (Thermo Fisher Scientific, Germany, cat. no. 32134), according to the manufacturer’s instruction.

**Legends**

**Supporting Figures**

**Supporting Figure 1: CD271 is a specific marker for quiescent and activated HSC in mouse livers.** Immunofluorescence (IF) staining using antibodies against desmin (a marker for all HSC) and Acta2 (a marker for activated HSC) coupled with RNAscope analysis for CD271 in mouse models of liver fibrosis. Septal fibrosis was induced using repeated intoxication with CCl_4_ treatment, and biliary fibrosis was modeled in Mdr2*KO* mice. The closeup images are shown in figure 1A and B. Scale bars are 200µm.

**Supporting Figure 2: CD271 is a specific marker for quiescent and activated HSC in human livers.** Tumor-free (NT) and fibrotic human liver samples were co-stained with RNAscope for CD271 and antibodies against Acta2 or RNAscope for CD271 and desmin. The closeup images are shown in figure 1E. Scale bars are 200µm.

**Supporting Figure 3:** **CD271 is expressed in desmin and Acta2 positive cells.** (A) IHC staining for serial sections of two human healthy livers shows that CD271 positive cells are co-localized with desmin or Acta2-expressing cells.  (B) Immunofluorescence staining of liver tissue revealed co-localization of CD271 (red) with Desmin and αSMA (green), indicating expression in hepatic stellate cells. Nuclei were counterstained with DRAQ5 (blue). Scale bar: 100 μm.

**Supporting Figure 4:** **Gating strategy for identification of HSC**. (A) dead cells are removed by gating out propidium iodide (PI) positive cells. (B) then debris is excluded using FSC abd SSC. C) Further gating strategy is used to remove autofluorescent cells using unstained APC vs Vio770 channel. The gated cells are used for further MACS flow cytometry analysis using specific HSC marker (desmin) and CD271 before and after autoMACs separation. Percentages indicate the gated cells.

**Supporting Figure 5: Isolation of liver cells from C57BL/6 and MdrKO mice.** (A) Livers of C57BL/6 mice of different ages are perfused and the 4 cell types are separated using GentleMACS system. (B) Cell numbers are presented as an average±SD of 3-4 mice per time point. (C) Livers of 4 months old Mdr2*KO* were dissected and dissociated in dissociation buffers upon applying the GentleMACs system. Then, using autoMACs separation and magnetic bead-labelled antibodies, namely CD271, F4/80 and CD146, were used to separate HSC, KC and LSEC, respectively. D) Cell numbers and viability are presented as an average ± SD of 4 Mdr2*KO* mice. (E) mRNA levels of Albumin, Desmin, Cd271, Cd11b and Lyve1 as a specific marker for HC, HSC, KC and LSEC, respectively.

**Supporting Table 1.** Primary antibodies used in the study

| **Antibody** | **Catalogue number/ Company** | **Application/ Dilution** | **Target Species** |
| --- | --- | --- | --- |
| Goat anti-NGFR/TNFRSF16 | AF1157/R&D systems | IF (tissue) / 1:100 | Mouse  Human |
| PE-conjugated CD271 (LNGFR) magnetic microbead | 130-118-793/ Miltenyi Biotec | AutoMACS/ 1:50  MACSQuant/ 1:100 | Mouse  Human |
| Mouse anti-CD271 (NGF receptor) | 14-9400-82/ Invitrogen | IHC/ 1:100 | Human |
| Rabbit anti-p75NTR (CD271) | #8238/ Cell Signaling Technology | ICC (Cells)/ 1:1000  WB / 1:1,000 | Mouse |
| FITC-conjugated rabbit anti-desmin | orb15501/ Biorbyt | MACSQuant/ 1:100 | Mouse |
| Rabbit anti-desmin | Ab32362/ Abcam | IF (tissue)/ 1:100  ICC (Cells)/ 1:250  IHC (tissue)/ 1:200  WB / 1:1000 | Mouse  Human |
| Mouse anti-human α-SMA | M0851/ DAKO | IF/ 1:100  IHC/ 1:500 | Mouse  Human |
| Rat anti-mouse F4/80 | 14-4801-85/ eBioscience | IF/ 1:100 | Mouse |
| Rat anti-mouse LYVE1 | sc-65647/ Santa Cruz Biotechnology | IF/ 1:100 | Mouse |
| Sheep Anti-human albumin | AB8940/ Abcam | IF/ 1:100 | Mouse  Human |
| Magnetic-microbead-conjugated-CD11b | 130-049-601/ Miltenyi Biotec | AutoMACS/ 1:50 | Mouse  Human |
| Magnetic-microbead-conjugated-CD146 | 130-092-007/ Miltenyi Biotec | AutoMACS/ 1:50 | Mouse  Human |
| Mouse anti-CD31 (PECAM-1) (89C2) Mouse mAb | #3528/ Cell Signaling Technology | WB/ 1:1000 | Mouse |
| Rabbit anti-F4/80 | sc-52664/ Santa Cruz Biotechnology | WB/ 1:750 | Mouse |
| Rabbit anti-Tubulin | ab4074-100/ Abcam | WB/ 1:1000 | Mouse |

IF: Immunofluorescence; IHC: Immunohistochemistry; ICC: Immunocytochemistry; WB: Western blotting; AutoMACS: automatic separation based on magnetic bead labelling; MACSQuant: Magnetic beads flow cytometry.

**Supporting Table 2.** Human liver cells used cell isolation from perfusion based method.

| **Patients** | | | | | **Total Viable HC (x10⁶)** | **Total Viable NPC (x10⁶)** | **HC before shipment** | | **NPC before shipment** | |
| --- | --- | --- | --- | --- | --- | --- | --- | --- | --- | --- |
| **Age (y)** | **Sex** | **Operation (Preliminary)** | **Diagnosis (Final)** | **Weight of Tissue Specimen (g)** |  |  | **Viable number (x10^6^)** | **Viability (%)** | **Viable number (x10^6^)** | **Viability (%)** |
| 74 | m | Hemihep. re. | CRLM (Z.n. LTX 2011) | 40.05 | 415 | 400 | 15 | 76% | 38 | ~95% |
| 60 | w | Liver resection Seg. II+III, atypical VI+VII | Hemangioma and CRLM | 23.52 | 298 | 581 | 15 | 80% | 46 | ~90% |
| 44 | w | Robot-assisted left-lateral resection | Hemangioma | 23.95 | 164 | 128 | 15 | 81% | 40 | 74% |
| 39 | m | Enucleation / Segment resection (right LL) | NET Met. | 19.38 | 334 | 394 | 15 | 74% | ~47 | ~83% |
| 39 | w | Completion after in-situ split | CRLM (state after in-situ split) | 18.02 n | 220 | 821 | 15 | 75% | ~49 | ~88% |
| 64 | w | Open segmental resection 7/8 | Adenofibroma (state after PVE) | 55.00 | 122 | 754 | 15 | 79% | ~50 | ~90% |
| 58 | w | Laparoscopic hand-assisted resection | Breast cancer metastasis (Mamma-Ca. Met.) | 25.25 | 146 | 868 | 16 | 70% | ~50 | ~90% |
| 60 | w | Robot-assisted resection (S6/7) | Hemangioma | 19.8 x | 208 | 80 | 15 | 83% | ~60 | ~82% |

**Supporting Table 3.** Secondary antibodies used in the study

| **Antibody** | **Catalogue number/ Company** | **Application/ Dilution** |
| --- | --- | --- |
| Cy3 conjugated donkey anti-rabbit IgG | 711-166-152/ Jackson ImmunoResearch | IF/ 1:200 |
| AlexaFluor®488 conjugated donkey anti-mouse IgG | 715-546-151/ Jackson ImmunoResearch | IF/ 1:200 |
| Cy3 conjugated donkey anti-goat IgG | 705-165-147/ Jackson ImmunoResearch | IF/ 1:200 |
| AlexaFluor488 conjugated donkey anti-rat IgG | 712-546-150/ Jackson ImmunoResearch | IF/ 1:200 |
| AlexaFluor555 conjugated donkey anti-sheep IgG | A-21436/ Invitrogen | IF/ 1:500 |
| HRP -conjugated goat anti-mouse IgG | P0447/ DAKO | IHC/ 1:200 |
| Biotinylated goat anti-mouse IgG | BA-9200/ Vector Laboratories | IHC/ 1:500 |
| HRP-conjugated Streptavidin | 016-030-084/ Jackson Immuno Research | IHC/ 1:500 |
| HRP -conjugated swine anti-rabbit IgG | P0217/ DAKO | IHC/ 1:200 |
| Anti-PE microbeads | 130-048-801/ Miltenyi Biotec | AutoMACS/ 1:9 |
| Goat anti-rabbit IgG-HRP | sc-2301/ Santa Cruz Biotechnology | WB/ 10:10.000 |
| Goat anti-mouse IgG-HRP | sc-2005/ Santa Cruz Biotechnology | WB/ 10:10.000 |

**Supporting Table 4:** RNAscope probes and controls

| **Probe** | **Supplier** | **Probe number** | **Species** |
| --- | --- | --- | --- |
| Hs-DES-C2 | Advanced Cell Diagnostic | 403041-C2 | Human |
| RNAscope® 3-plex Positive Control Probe - Hs | Advanced Cell Diagnostic | 320861 Human positivecontrol probe for RNAscope® Multiplex Fluorescent Assay | Human |
| RNAscope® 3-plex Negative Control Probe | Advanced Cell Diagnostic | 320871 for RNAscope® Multiplex Fluorescent Assay, RNAscope® Negative control probe DapB (of Bacillus subtilis strain) | Human/Mouse |
| RNAscope™ Probe- Mm-Ngfr | Advanced Cell Diagnostic | 494261- C1 | Mouse |
| RNAscope 3-Plex Negative Control Probe | Advanced Cell Diagnostic | 320871 RNAscope 3-Plex Negative Control | Mouse |
| RNAscope Probe Hs-NGFR (CD271)-C1 | Advanced Cell Diagnostic | 406331-C1 | Human |
| RNAscope positive Control Probe-Mm-Ppib | Advanced Cell Diagnostic | 313911 | Mouse |
| RNAscope Control slides | Advanced Cell Diagnostic | 310045- Human Hela Cell Pellet | Human |
| RNAscope control slides | Advanced Cell Diagnostic | 310023- Mouse 3T3 Cell Pellet | Mouse |

**Supporting Table 5.** Sequences of primer pairs used for real time PCR

| **Gene** | **Species** | **Forward (5’-3’)** | **Reverse (5’-3’)** |
| --- | --- | --- | --- |
| Acta2 | Mouse | TTCGCTGTCTACCTTCCAGC | GAGGCGCTGATCCACAAAAC |
| Albumin | Mouse | TCCTGATTGCCTTTTCCCAGTATCT | GCCAGTTCACCATAGTTTTCACGGA |
| Cd11b | Mouse | AAACCACAGTCCCGCAGAGA | CGTGTTCACCAGCTGGCTTA |
| Cd271 | Mouse | TGCCTGGACAGTGTTACGTTC | CAGTCTCCTCGTCCTGGTAGT |
| Col1α1 | Mouse | ACGTGGAAACCCGAGGTATG | TTGGGTCCCTCGACTCCTAC |
| Desmin | Mouse | TACACCTGCGAGATTGATGC | ACATCCAAGGCCATCTTCAC |
| Lyve1 | Mouse | CAGCACACTAGCCTGGTGTTA | CGCCCCATGATTCTGCATGTAGA |
| Ppia | Mouse | GAGCTGTTTGCAGACAAAGTC | CCCTGGCACATGAATCCTGG |
| Acta2 | Human | AAGAGCATCCGACACTGCTGAC | AGCACAGCCTGAATAGCCACATAC |
| Albumin | Human | ACTGCATTGCCGAAGTGGA | GCAGCACGACAGAGTAATCAGGA |
| CD146 | Human | AACACAGTGGGCGCTATGAA | AACTCGAGGTCCTGGCTACT |
| CD271 | Human | AACCTCATCCCTGTCTATTG | GTTGGCTCCTTGCTTGTT |
| CD11B | Human | AAGATGCCCACTGAGGAATG | TCTGGAAAGGGAGACTTTTCAC |
| Col1α1 | Human | CGGACGACCTGGTGAGAGA | CATTGTGTCCCCTAATGCCTT |
| Desmin | Human | AACCAGGAGTTTCTGACCACG | TTGAGCCGGTTCACTTCGG |
| Ppia | Human | AGGGTTCCTGCTTTCACAGA | CAGGACCCGTATGCTTTAGG |

**Supporting Table 6. Comparison of the proposed protocol with Mohar et al., 2015 method.** The two protocols offer distinct strategies for isolating NPCs from the liver, differing primarily in their digestion steps, enrichment techniques, and scalability. The Mohar et al. protocol is a well-established, manual, high-throughput. The Dropmann et al. protocol is a semi-automated upgradable method that incorporates MACS with a novel marker for superior purity and versatility across species.

| Category | Mohar et al., 2015 | Proposed protocols |
| --- | --- | --- |
| Main goal | Provide a reproducible, classical protocol for isolating liver non-parenchymal cells (NPCs). | Improve liver cell isolation using CD271-based sorting and semi-automation of main liver cells with possible upgrading to include other liver cells and rare cell types |
| Liver dissociation method | Manual portal-vein collagenase perfusion, mechanical dissociation. | Automated or semi-automated dissociation workflow using gentleMAC Octo Dissociator or vena cava perfusion. |
| Cell enrichment | Density gradient to separate hepatocytes and NPCs. | Low-high speed centrifugation to separate hepatocyte and NPCs. NPCs were enriched using density gradient and were isolated based antibody-magnetic beads using CD271, CD11b and CD146 for HSC, KC and LSEC populations. |
| Cell types targeted | Broad NPCs: LSECs, Kupffer cells, quiescent HSCs, lymphocytes. | Hepatocytes and 3 NPC types and upgradable |
| Downstream purification | FACS or MACS depending on cell type; detailed gating strategies provided. | Sorting is not required since we used autoMACs separator to enrich the NPC fractions based on cell type specific marker. |
| Detail level | Very detailed step-by-step instructions, notes, troubleshooting, QC examples (no info on cell yield). | High-level focus on innovation, efficiency with detailed levels for yield, purity, and viability as well as cell functionality. |
| Instrumentation needs | Standard perfusion setup, centrifuges, FACS/MACS. | Requires antibody panels, semi-automation equipment. |
| Hands-on time | High manual, operator-dependent, slower throughput. | Lower semi-automation likely improves speed and reproducibility. |
| Reproducibility | Moderate; relies heavily on operator skill (perfusion quality). | High; automation and marker-based sorting. |
| Yield / purity | Good purity via gradient + FACS; QC by qPCR included. No cell numbers were reported | Improved yields/purity. |
| Applicability across conditions | Valid for healthy and parasitic infected mouse livers (C57BL/6J). | Validated as an age, strain, species, disease state independent method. |
| Cost | Low–moderate (collagenase, gradients, standard lab materials). | Moderate- Higher (antibodies and automation consumables). |
| Best use case | When robust, broadly applicable, cost-effective NPC isolation is needed. | When standardization, high throughput, and simultaneous multi-cell recovery are priorities. |

**References**

1. Pradere JP, Kluwe J, De Minicis S, Jiao JJ, Gwak GY, Dapito DH, et al. Hepatic macrophages but not dendritic cells contribute to liver fibrosis by promoting the survival of activated hepatic stellate cells in mice. Hepatology (Baltimore, Md). 2013;58(4):1461-73. doi: 10.1002/hep.26429. PubMed PMID: 23553591; PubMed Central PMCID: PMC3848418.

2. Godoy P, Widera A, Schmidt-Heck W, Campos G, Meyer C, Cadenas C, et al. Gene network activity in cultivated primary hepatocytes is highly similar to diseased mammalian liver tissue. Arch Toxicol. 2016;90(10):2513-29. doi: 10.1007/s00204-016-1761-4. PubMed PMID: 27339419; PubMed Central PMCID: PMC5043005.

3. Hammad S, Ogris C, Othman A, Erdoesi P, Schmidt-Heck W, Biermayer I, et al. Tolerance of repeated toxic injuries of murine livers is associated with steatosis and inflammation. Cell Death Dis. 2023;14(7):414. Epub 20230712. doi: 10.1038/s41419-023-05855-4. PubMed PMID: 37438332; PubMed Central PMCID: PMC10338629.

4. Shriki A, Lanton T, Sonnenblick A, Levkovitch-Siany O, Eidelshtein D, Abramovitch R, et al. Multiple Roles of IL6 in Hepatic Injury, Steatosis, and Senescence Aggregate to Suppress Tumorigenesis. Cancer Res. 2021;81(18):4766-77. Epub 20210611. doi: 10.1158/0008-5472.CAN-21-0321. PubMed PMID: 34117031.
