# Supplementary material for: Semi-automated isolation of parenchymal and non-parenchymal liver cells from mice and humans with enhanced stellate cell fraction
Source: Cell Biosci. 2026 Mar 14;16:45. doi: 10.1186/s13578-026-01554-7 (PMC13123112; doi:10.1186/s13578-026-01554-7)

Supporting Fig 1

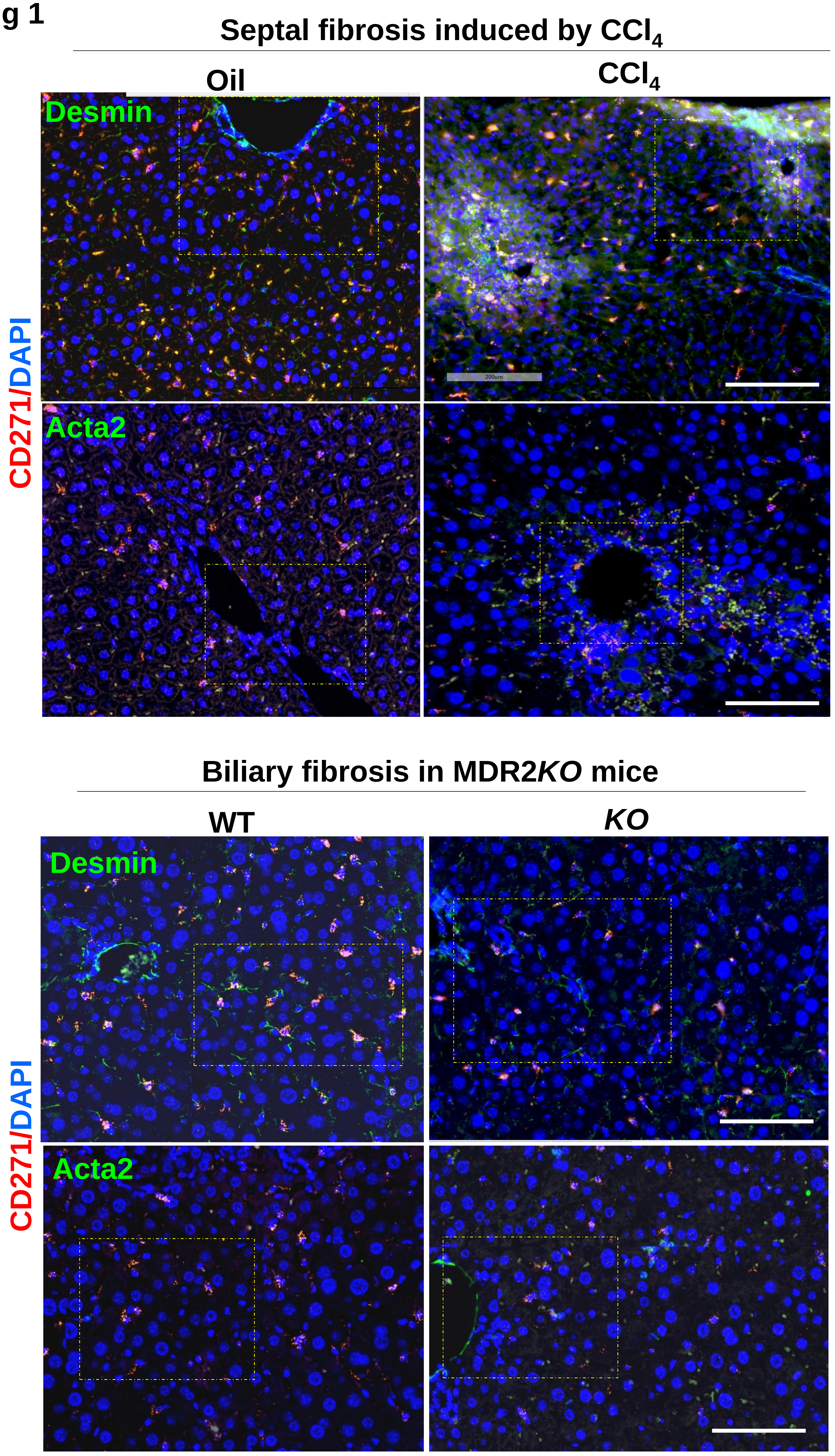

Supporting Fig 2

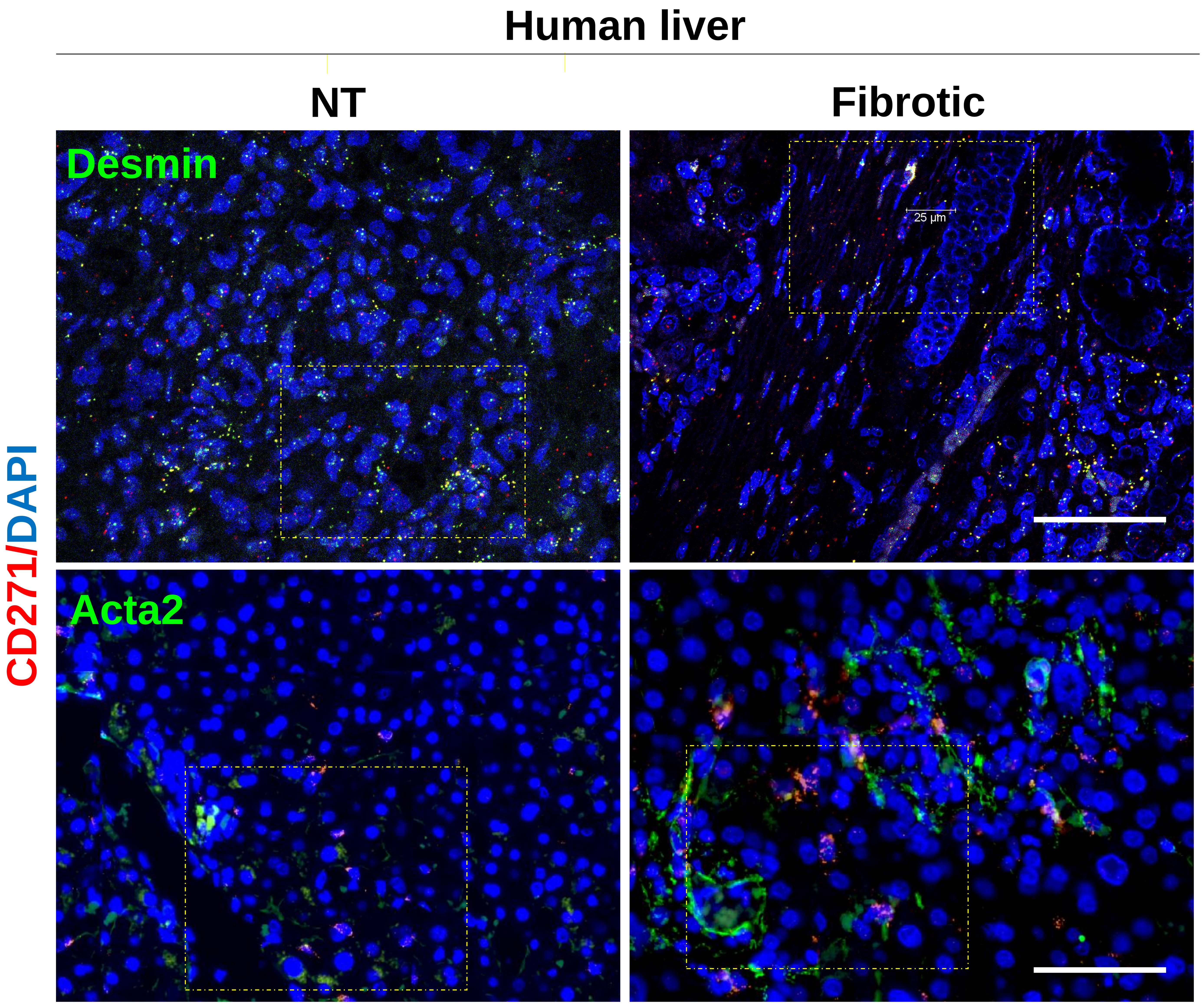

Supporting Fig 3

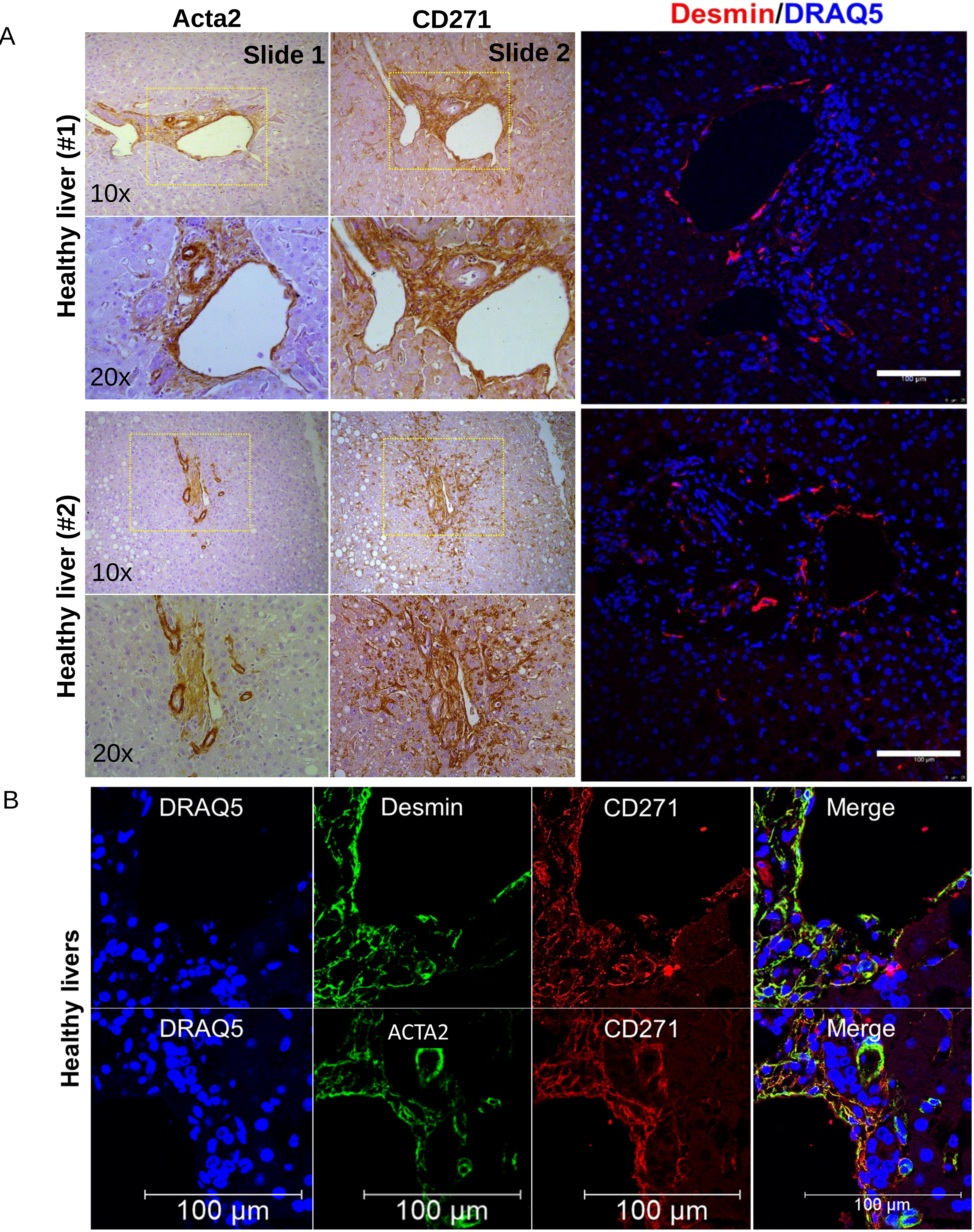

Supporting Figure 4

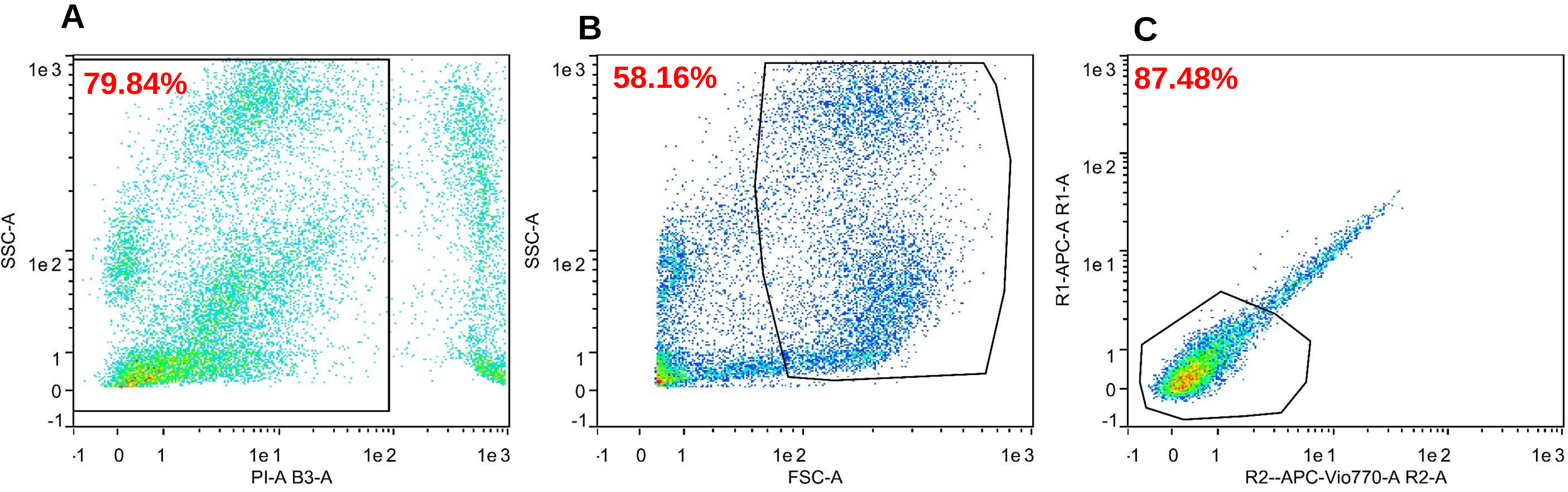

A

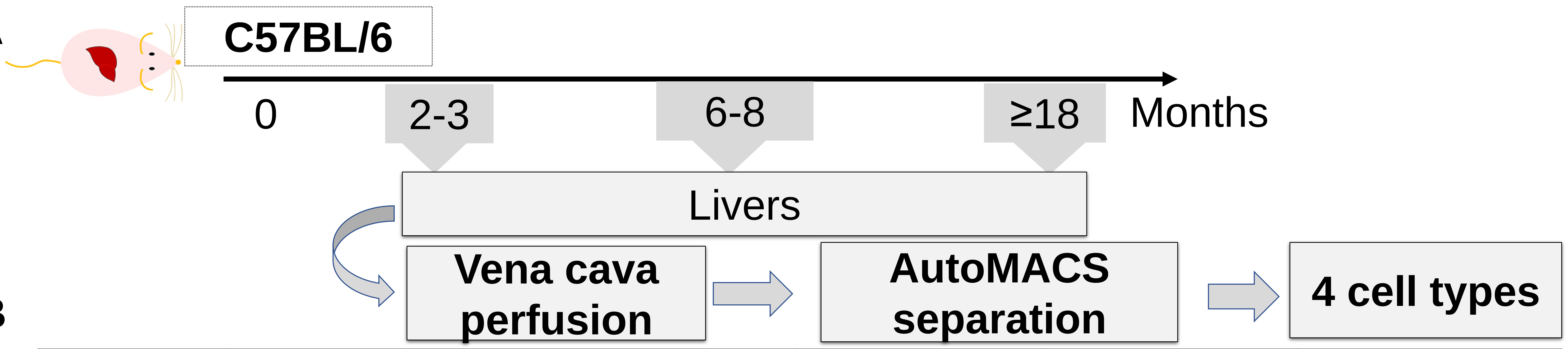

B

Age (m)		HC	HSC	KC	LSEC
**2-3**	**Cell yield (No.±SD)**	35.8±2.1x 10^6	122.5±44x 10^3	63.8±19.6x 10^4	1.99±0.23x 10^6
	**Viability (%±SD)**	90.8±6.7	86.2±8.1	91.1±7.4	88.4±7.5
**6-8**	**Cell yield (No.±SD)**	31.8±7.7x 10^6	892.1±69x 10^3	151.0±53x 10^4	2.22±0.30x 10^6
	**Viability (%±SD)**	95.2±7.1	89.7±8.1	92.6±7.4	89.9±6.6
**≥18**	**Cell yield (No.±SD)**	10.7±6.2x 10^6	309±175x 10^3	97.3±16.5 x10^4	1.20±0.13x 10^6
	**Viability (%±SD)**	86.2±7.4	85.6±6.2	88.2±8.9	86.8±9.8

C

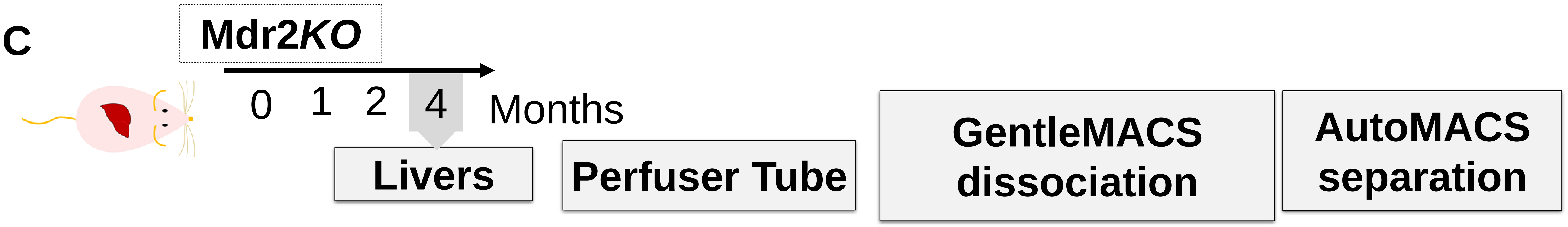

D

	HC	HSC	KC	LSEC
**Cell yield (No.±SD)**	4.5x$10^{5\pm}$2.09	100±50x10^3	2.8±0.82x10^6	2.0±0.27x10^6
**Viability (%±SD)**	85,9 ± 8.2	88.8±9.2	89.2±9.8	87.9±11.3

E

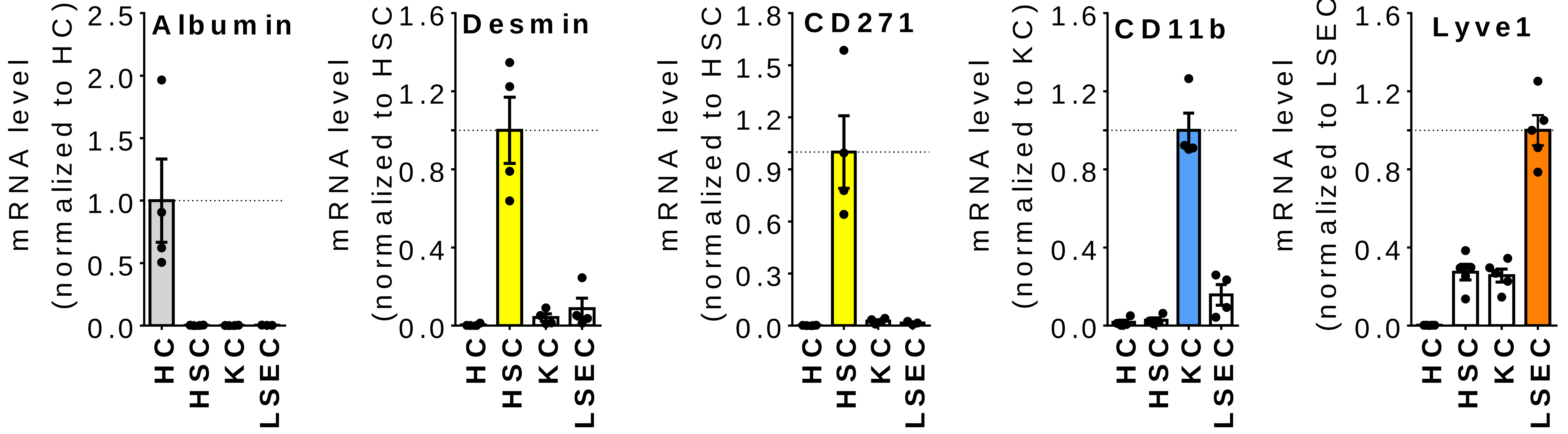

Supplement: Supplementary file 2 — Supplementary Material 2. [file 13578_2026_1554_MOESM2_ESM.pdf]
